# Medical Opinion and Movement

**Published:** 1907-05-11

**Authors:** 


### Medical Opinion and Movement.

The new Workmen's Compensation Act will affect a much larger proportion of the community than any preceding Act of the same nature, and the immediate effect will undoubtedly be an enormous increase in the extent of insurance against all manner of accidents and injuries. This should result in considerable financial advantage to members of the medical profession, as it should secure to them adequate remuneration for services, for which, in other circumstances, payment is often scanty or altogether absent. Nevertheless, unless medical practitioners show a united stand in regard to fees due to them under the Act they may run the serious risk of being exploited, and of finding their claims successively repudiated, by the insurance company, the employer, and the employé. The matter was discussed recently at a meeting of the Newbury and District Medical Society, and the following resolutions were unanimously carried: "That this meeting is of opinion that the certificates issued under the Workmen's Compensation Act by any insurance company, which are required to be filled in at the expense of the injured workman, and stating the probable duration of disablement, should not be signed by any member of this society." "That no certificate be filled up on behalf of the employer under the above Act for a less fee than one guinea."

The University of New Zealand, which, like most Colonial universities, is keeping abreast of the times, has embarked on a novel scheme which will appeal to all who are interested in educational developments. By instituting the degree of "Bachelor in Original Research" (presumably to be abbreviated into B.R.) it is affording students who are specially interested in one or other department of study a golden opportunity to work wholeheartedly at their speciality. A three years' course is necessary to obtain the degree. During the first two years the candidate is required to pass preliminary examinations in the subjects or departments he wishes to take up, together with such allied subjects as may be deemed desirable by the Faculty to which he attaches himself, but during the third year he is free to specialise. The degree itself is granted on a thesis dealing with some special work, the result of the third year's separate study, and referred to a special board of examiners for adjudication. No restrictions, apparently, are placed on candidates as to what lines they should follow, and it is reasonable to expect that a large number of those who avail themselves of the new regulations will embark on scientific research. The opportunities for original work in medical science are particularly good in New Zealand, and it will be interesting to see whether or not the new departure of the University will further such work.

The April number of *The Paris Medical Journal* contains the following announcement by the editors, Dr. E. L. Gros and Dr. A. A. Warden, physician to the Hertford British Hospital, Paris: "The Editors regret that this number, with which they had hoped to inaugurate a new year, must be the last. With the best of goodwill they find it impossible to reconcile the claims of the paper with the increasing duties of private practice." The announcement will occasion much regret, and we earnestly hope that some means will be found to continue the existence of our contemporary. We venture to think that the "Paris Medical Journal" has done much to bring medical practitioners in England into touch with their professional colleagues in France. Always ably edited, always interesting in its subject-matter and dignified in its tone, this journal is one to which many on this side of the Channel have become attached. It gave us the newest and best in French medical methods of diagnosis and treatment, and its strong staff—representing in France such distinguished teachers as Dieulafoy, Dejèrine, Pinard, Débove, and Pozzi, and here in England Professor Osler, Sir A. Wright, Mr. D'Arcy Power, Mr. Mayo Robson, and Sir Alfred Fripp—was an assurance of excellence were such assurance not rendered entirely unnecessary by the quality of the journal itself. To Drs. Gros and Warden the profession, both here and in France, owes a debt of gratitude and praise for the attention they have given in the midst of their professional duties to the editorial work of a bilingual journal whose cessation is a matter for sincere regret.

Mr. W. Arbuthnot Lane has lately written somewhat extensively on his method of treatment of chronic constipation by short-circuiting the intestines so as to put the large intestine altogether out of action. The method has not so far been received very favourably by the profession. We have not heard of any other surgeon advocating or employing this treatment, and it does not seem likely that such heroic measures will receive anything like general adoption, except, perhaps, in extreme cases such as have been graphically described by Mr. Lane. But, personally, we are disposed to think that cases in which definite pathological changes have taken place in the lower bowel owing to severe constipation extending over many years, are not so common as Mr. Lane would lead us to believe. Nevertheless, Mr. Lane has already found reason to perform his operation several times, and he certainly secured a triumph for his operative skill by showing eleven patients (10 females, 1 male) on whom he had carried out the operation to a large number of medical men at Guy's Hospital on April 28. The patients themselves bore witness that previous to the operation they had suffered from wasting and pain resulting in inability to work, and that since the operation they had regained health and strength, and were able to take their ordinary food and suffered from no inconvenience. In some instances the operation had been performed three years ago.

